# Prospective trial of different antimicrobial treatment durations for presumptive canine urinary tract infections

**DOI:** 10.1186/s12917-021-02974-y

**Published:** 2021-09-06

**Authors:** Fergus Allerton, Koen B. Pouwels, Julien Bazelle, Sarah Caddy, Andria Cauvin, Luisa De Risio, James Swann, James Warland, Andrew Kent

**Affiliations:** 1 Willows Veterinary Centre and Referral Service; part of Linnaeus Veterinary Limited, Highlands Road, Shirley, Solihull UK; 2 Health Economics Research Centre, Nuffield Department of Population Health, University of Oxford, Oxford, UK; 3 NIHR Health Protection Research Unit in Healthcare Associated Infections and Antimicrobial, Oxford, UK; 4 Resistance at University of Oxford in partnership with Public Health England, Oxford, UK; 5 Davies Veterinary Specialists; part of Linnaeus Veterinary Limited, Manor Farm Business Park, Higham Gobion, Hitchin, UK; 6 Cambridge Institute for Therapeutic Immunology and Infectious Disease, Jeffery Cheah Biomedical Centre, Puddicomb Way, Cambridge Biomedical Campus, Cambridge, UK; 7 Beechley Farm, Park Hall Road, Gosfield, Essex UK; 8 Linnaeus Veterinary Limited, Friars gate, Shirley, Solihull, UK; 9 Columbia Stem Cell Initiative, Columbia University, 650 West 168th Street, NY 10032 New York, USA; 10 Wellcome-MRC Cambridge Stem Cell Institute, Jeffrey Cheah Biomedical Centre, University of Cambridge, Puddicombe Way, Cambridge, UK; 11 Willows Veterinary Centre and Referral Service; part of Linnaeus Veterinary Limited, Highlands Road, Shirley, Solihull UK

**Keywords:** Antimicrobial stewardship, Antimicrobial resistance, duration, Urinary tract infection, Canine, Duration-response curve

## Abstract

**Background:**

Avoidance of unnecessary antimicrobial administration is a key tenet of antimicrobial stewardship; knowing the optimal duration of therapy obviates over-treatment. However, little research has been performed to establish course lengths for common canine infections. In clinical practice, antimicrobial therapy is frequently prescribed in dogs presenting lower urinary tract signs (haematuria, pollakiuria and dysuria/stranguria). The proposed length of treatment in International Consensus guidelines has decreased with each iteration, but these recommendations remain arbitrary and largely extrapolated from experience in people.

**Methods:**

The objective of this prospective, multi-centre study is to find the shortest course duration that is non-inferior to the standard duration of 7 days of amoxicillin/clavulanate in terms of clinical outcomes for female dogs with lower urinary tract signs consistent with a urinary tract infection. An electronic data capture platform will be used by participating veterinarians working in clinical practice in the United Kingdom. Eligible dogs must be female, aged between 6 months and 10 years and have lower urinary tract signs of up to seven days’ duration. Enrolment will be offered in cases where the case clinician intends to prescribe antimicrobial therapy. Automatic pseudo-randomisation to treatment group will be based on the day of presentation (Monday-Friday); all antimicrobial courses will be completed on the Sunday after presentation generating different treatment durations. Follow-up data will be collected 1, 8 and 22–26 days after completion of the antimicrobial course to ensure effective safety netting, and to monitor short-term outcome and recurrence rates. Informed owner consent will be obtained in all cases. The study is approved by the Ethical Review Board of the University of Nottingham and has an Animal Test Certificate from the Veterinary Medicine’s Directorate.

**Discussion:**

This study has been designed to mirror current standards of clinical management; conclusions should therefore, be widely applicable and guide practising veterinarians in their antimicrobial decision-making process. A duration-response curve will be created allowing determination of the optimal treatment duration for the management of female dogs with lower urinary tract signs. It is hoped that these results will contribute valuable information to improve future antimicrobial stewardship as part of a wider one-health perspective.

**Supplementary Information:**

The online version contains supplementary material available at 10.1186/s12917-021-02974-y.

## Background

The therapeutic value of antimicrobials has been amply demonstrated over the past century both in lives saved and suffering alleviated. However, the continued success of antimicrobial therapy now hangs in the balance due to developing antimicrobial resistance (AMR). AMR is estimated to cause over 30,000 deaths and almost 900,000 disability-adjusted life-years (DALYs) a year in the EU and EEA alone. This estimated burden of AMR – excluding tuberculosis - is similar to the annual cumulative burden of influenza, tuberculosis and human immunodeficiency virus (HIV) [1]. In recognition of the role of antimicrobial use in driving AMR, antimicrobial stewardship initiatives have been developed to try to guide prescribers to optimise their antimicrobial use. For veterinarians these tools include the British Small Animal Veterinary Association (BSAVA)/Small Animal Medicine Society (SAMSoc) PROTECT ME poster, Federation of European Companion Animal Veterinary Associations (FECAVA) guidelines and the Danish Antimicrobial Use Guidelines for Companion Animal Practice [2]. Recommendations are derived from the limited evidence available in the literature and consensus statements such as the guidelines produced by the International Society for Companion Animal Infectious Diseases (ISCAID) [3–5].

Bacterial urinary tract infection (UTI), whether presumptive or confirmed, is a common motive for consultation in small animal practice and accounts for 6–12 % of antimicrobial prescriptions in dogs [6–9]. A lifetime prevalence of UTI of 14 % has been reported in dogs [10] with a marked predisposition among females [10, 11] due to the shorter urethral length and increased incidence of bacteriuria compared to males [12]. Clinical suspicion of a UTI is based on the presentation of compatible lower urinary tract signs (pollakiuria, dysuria/stranguria and haematuria). However, these signs are not pathognomonic for the presence of infection, with only 46 to 65 % of dogs with one or more clinical signs of lower urinary tract disease having infection confirmed at culture [13–15].

The previous classification system for canine UTIs, extrapolated from human medicine, described uncomplicated or complicated infections [16]. However, prolonged treatment with an antimicrobial as recommended for a complicated UTI is not always warranted. Recently, a new nomenclature system has been introduced, defining all initial or infrequent (< 3 episodes of cystitis in the preceding year) presentations in non-gravid females as ‘canine sporadic cystitis’ even if urinary tract abnormalities or comorbidities are present [5]. The emphasis in the new terminology is on the presence of inflammation and consequent compatible clinical signs [5]. Subclinical bacteriuria is thereby excluded; it is recommended to avoid antimicrobial therapy in the absence of lower urinary tract signs [5].

Bacterial culture and antimicrobial susceptibility testing can be performed to demonstrate bacterial involvement and to optimise antimicrobial selection. However, empirical therapy is typically commenced pending culture results. While antimicrobial stewardship guidelines and the ISCAID guidelines advocate submitting samples for culture in all cases of sporadic cystitis, it is recognised that this may not occur routinely in practice. In the PROTECT ME guidelines, culture is strongly advised but not deemed essential for cases of uncomplicated cystitis. To the authors’ knowledge, the proportion of dogs treated for suspected UTI that have a confirmatory culture has not been determined. Research in human medicine found that cultures were requested in only 10–40 % of cases of suspected UTI despite high levels (74–94 %) of antimicrobial use [17–19]. In one study, out of 1148 cats treated with cefovecin (157 with clinical signs relating to the urinary tract), bacterial culture was performed only 4 times [20]. While these examples illustrate a stark divergence between guideline recommendations and standard practice, they offer a representative reflection of the decision-making associated with antimicrobial prescription.

According to the latest data from the European Medicines Agency, amoxicillin/clavulanate is the most commonly prescribed antimicrobial tablet in Europe [21]. It accounted for 44 % of antimicrobial prescriptions in first-opinion practices in the UK [22]. Amoxicillin is a time-dependent antimicrobial with a broad spectrum of action against Gram positive and Gram negative bacteria that commonly cause UTI in dogs. In recent studies in the UK and USA, approximately 50–75 % of pathogen isolates were sensitive to amoxicillin/clavulanate with rates of up to 90 % in uncomplicated cases [23, 24]. These figures are likely to underestimate biological sensitivity as amoxicillin is concentrated in urine potentially overcoming some of the resistance of beta-lactamase producing bacteria [5]. Urine drug concentrations obtained following administration of standard doses of antimicrobials have been published for healthy animals [25]. Susceptibility of an identified uropathogen may be anticipated if the achieved concentration exceeds the minimum inhibitory concentration (MIC) in vitro.

Amoxicillin/clavulanate, used for 10–14 days to treat confirmed bacterial UTI in dogs, had a clinical cure rate of approximately 70–80 % and microbiological cure rates of 85–90 % [26, 27]. Short antimicrobial regimens of 3 days of either enrofloxacin or trimethoprim/sulphonamide were not inferior to either 14 days of amoxicillin/clavulanate or 10 days of cephalexin respectively in dogs with bacterial UTI [27, 28]. These studies, demonstrate that effective clinical and microbiological cure may be achieved in most cases of sporadic cystitis with short courses of antimicrobial therapy, at least for these particular antimicrobials. The recommended duration for treatment stated in the marketing authorisations for amoxicillin/clavulanate of 5–7 days is not supported by published evidence in the veterinary literature. Despite the commonness of this condition, robust data to inform the optimal duration of antimicrobial treatment are still lacking [29].

An emerging dogma in human medicine, in relation to duration of antimicrobial treatment, asserts that shorter is better [30, 31]. Nevertheless, general practitioners often prescribe longer courses than the duration recommended in national guidelines [32]. Administration of antimicrobials beyond what is necessary to achieve clinical resolution risks fostering AMR at remote sites (including the microbiome) due to sustained antimicrobial exposure and thus selective pressure favouring resistance. In human patients with pneumonia, excess treatment was not associated with improved outcomes (lower death rates or risks of recurrence) but did incur a 5 % increase in the likelihood of drug-related adverse events [33].

Mirroring this principle, the consensus recommendation from the latest ISCAID guidelines advocates a duration of antimicrobial treatment of just 3–5 days for sporadic cystitis [5]. Standardised course lengths (often arbitrarily based on multiples of 5 or 7 days) offer less flexibility than a tailored approach of treating until (or just beyond) clinical cure. However, such a dynamic approach relies heavily on the capacity of an owner to accurately interpret their pet’s clinical signs to inform their decision to stop treatment. Robust evidence supporting shorter antimicrobial course lengths is required to optimize antimicrobial therapy in dogs.

In summary, findings from past research and extrapolation from current practice in human medicine provides support for the adoption of shorter antimicrobial courses. However, robust evidence is required to determine the optimal duration of treatment for sporadic cystitis so that veterinarians can manage these patients with confidence. The rationale of the current study is to provide meaningful data relevant to current standard practice and to guide a single aspect of antimicrobial prescription. Since an intention to treat with antimicrobials is a pre-requisite inclusion criterion, the study is focused on comparing clinical outcomes to different durations of therapy.

The primary objective of the study is to estimate the shortest antibiotic treatment duration (out of 3, 4, 5, 6 days) that is non-inferior to 7 days of treatment with amoxicillin/clavulanate in terms of clinical cure among female dogs with lower urinary tract signs consistent with a urinary tract infection. A second aim is to evaluate recurrence rates within 4 weeks of starting antimicrobial therapy across these different course durations. Through estimation of a duration-response curve using fractional polynomial logistic regression, the optimal treatment duration for the management of presumptive bacterial UTIs in female dogs may be determined [34–36]. The optimal duration is here defined as the shortest duration non-inferior to the control (maximum) duration within a risk difference margin of 10 % [36] Bootstrapping can be used to estimate the confidence intervals around the optimal duration at the pre-specified non-inferiority margin (10 %). This analysis approach has been shown to have good operating characteristics (type-1 and type-2 errors) under a wide range of true duration-response curves [36]. The main advantage of assigning female dogs with lower urinary tract signs consistent with sporadic cystitis to a number of duration arms and modelling the duration-response curve is that one does not have to select one arbitrary shorter duration to test against the standard duration [34–36]. This information can support future antimicrobial stewardship recommendations and help avoid unnecessary antimicrobial use.

## Methods and study design

### Recruitment

Veterinarians working in clinical practice in the United Kingdom have been informed about this trial through different media including a call for involvement in the study in a veterinary journal [37], presentation of the protocol at the Small Animal Society’s autumn meeting and promotion on the society website. Each participating veterinarian has been provided with a unique login to the electronic data capture system (see details below) and a package of documents relating to the study (including client consent forms, a user guide covering data entry, a summary of all the phases of the study and copies of the inclusion and exclusion criteria). Participating veterinarians are responsible for patient recruitment based on pre-defined eligibility criteria.

### Investigational animals

#### Informed consent

Only dogs that satisfy all of the inclusion criteria and to whom none of the exclusion criteria apply will be enrolled. Enrollment to the study will only be possible for dogs presenting to the participating veterinarian between Monday and Friday inclusive. All owners must provide written informed consent to participate in the study and also agree to fulfil all requirements of the study. A written version of the owner consent form and an information sheet for owners will be presented to the owner at initial consultation and will be verbally described by the veterinarian. The owner consent form and information sheet detail the nature of the study, what it will involve for the participant, the known side effects and any risks involved in taking part. It is clearly stated that owners are able to withdraw their dog from the study at any time and that withdrawal will not affect the care of their pet. The owner will be allowed time to consider the information and will be given the opportunity to question the participating veterinarian prior to enrolment. A copy of the signed owner consent form will be retained by the owner and the original retained at the practice of the participating veterinarian.

#### Inclusion and exclusion criteria

All dogs participating in this study are pets or working dogs. All dogs must be female, at least 6 months of age and less than 10 years of age on the day of enrollment. Dogs presented with acute onset of one or more of the following lower urinary tract clinical signs (pollakiuria, dysuria/stranguria or haematuria) are eligible for inclusion in the study as long as the case veterinarian intends to prescribe antimicrobials.

The dog may not be included in the study if a non-bacterial cause of the clinical signs is identified (e.g. urolithiasis or neoplasia of the urogenital tract), if there is suspicion of upper urinary tract involvement (presence of abdominal/lumbar pain, pyrexia (rectal temperature > 39.5 °C)), if the dog has a history of recurrent UTIs (previous lower urinary tract signs reported in the past 28 days, three or more episodes of clinical bacterial cystitis in the preceding 12 months or two or more episodes in the preceding 6 months), if the dog is being currently treated with corticosteroid therapy, insulin, trilostane or thyroxine supplementation, if the dog has received antimicrobials for any reason in the past 14 days, if there is known intolerance to amoxicillin/clavulanate or if the owners are unable to administer medications twice daily throughout treatment period.

### Study design

This trial is a prospective, quasi-randomised controlled trial to test the efficacy of shorter durations (3,4,5 and 6 days) compared to a longer standard duration of 7 days of antimicrobial treatment for female dogs with presumed UTI. Similar to the recently proposed DURATION design, the trial will include multiple duration arms which will be used to estimate a duration-response curve through fractional polynomial logistic regression. The duration-response-curve can subsequently be used to identify the optimal duration as proposed in Quartagno et al. [36] and described in the ‘data analysis’ section. A non-treatment control group is not included as it would not be ethical to deprive patients of antimicrobial treatment. Both the patient enrolment and follow-up appointments for individual cases will be held in the same clinic. Data analysis will be performed by study investigators in their respective institutions. Throughout the trial, participants will be kept in their home environment where reporting of clinical signs will occur.

The monitoring site for this trial is Willows Veterinary Centre and Referral Service, which will oversee the logistical progression of the study under the supervision of the Chief Investigator. This study is conducted in accordance with veterinary Good Clinical Practice (GCP) guidelines ((i) International Cooperation on Harmonisation of Technical Requirements for Registration of Veterinary Products (VICH) GL9 Good Clinical Practices (July, 2001), (ii) The European Agency for the Evaluation of Medicinal Products (EMEA) Guideline on statistical principles for veterinary clinical trials, EMEA/CVMP/816/00-FINAL (June 5, 2002)). The study is approved by the Ethical Review Board of the University of Nottingham (REF 3007 191,023).

### Electronic data collection platform

Data will be submitted through an Electronic Data Capture platform (CASTOR™) [38]. This platform can be used by multiple clinics at the same time and is updated live. The use of this platform facilitates compliance with medical data privacy, security, and GCP regulations. Participating veterinarians will complete study forms to record information about the dog on the day of enrolment, the Monday after completion of the antimicrobial course and 3 weeks later. Dogs will be automatically assigned a unique study number for identification purposes ensuring that all follow-up information from the participating veterinarian or from the owner is added to the specific clinical record. Web links to questionnaires will be automatically distributed to the owners via email 7 days after completion of the antimicrobial course and 28 days after initial presentation. Owners without access to the internet will be given a paper version of the same questionnaire or it may be completed on their behalf by the participating veterinarian using information provided by the owner.

### Initial presentation

Any eligible dog presenting to participating veterinarians with compatible lower urinary tract signs will be considered for enrolment in the trial. Support material, including details of the inclusion and exclusion criteria, has been prepared for participating veterinarians (see supplementary information). A complete clinical history will be taken and physical examination performed by the participating veterinarian. Demographic data including neuter status, bodyweight, body condition score, age and breed will be entered into a study form on the electronic data capture platform. The presence (and duration) of macroscopic haematuria (presence of red blood cells in the urine), pollakiuria (increased frequency of urination) and dysuria/stranguria (straining and difficulty to pass urine) will also be recorded.

Any additional tests or investigations (e.g. urinalysis or abdominal imaging) will be offered and completed at the participating veterinarian’s discretion before treatment commences. For inclusion in the study, cytological evidence supporting bacterial cystitis (e.g. pyuria, bacteriuria) or positive bacterial culture results are not required as these tests are not deemed mandatory examinations in standard clinical practice at first presentation. However, should these investigations be performed, the information will be entered into the dog’s electronic record. Dogs with evidence of a non-bacterial cause for the clinical signs will be excluded from the study.

At this consultation informed consent will be obtained from the owner after the initial eligibility check is completed. Dogs enrolled in the study will be prescribed the appropriate duration of antimicrobial treatment (number of doses) determined by the day of presentation to the clinic (Table 1). For the purposes of this study, only authorised preparations of amoxicillin-clavulanate will be prescribed as antimicrobial therapy. An Animal Test Certificate (ATC-S-130) has been obtained from the Veterinary Medicines Directorate covering the use of formulations of amoxicillin-clavulanate with a marketing authorisation in the United Kingdom for a variable treatment duration. In accordance with the marketing authorisations, a set dose of 12.5 mg/kg PO q12h should be rounded up to the nearest possible tablet size or fraction that can be administered. Tablets may be divided by the owner, if appropriate for the particular product dispensed according to instructions in the marketing authorisation. Tablets may be crushed for administration if necessary to ensure compliance.

**Table 1:** Schedule of antimicrobial treatment

Day/time of presentation	Monday	Tuesday	Wednesday	Thursday	Friday	Saturday	Sunday	Total doses
Mon. AM	*	*	*	*	*	*	*	14
Mon. PM	*	*	*	*	*	*	*	13
Tues. AM		*	*	*	*	*	*	12
Tues. PM		*	*	*	*	*	*	11
Wed. AM			*	*	*	*	*	10
Wed. PM			*	*	*	*	*	9
Thur. AM				*	*	*	*	8
Thur. PM				*	*	*	*	7
Fri. AM					*	*	*	6
Fri. PM					*	*	*	5

**Fig. 1:**
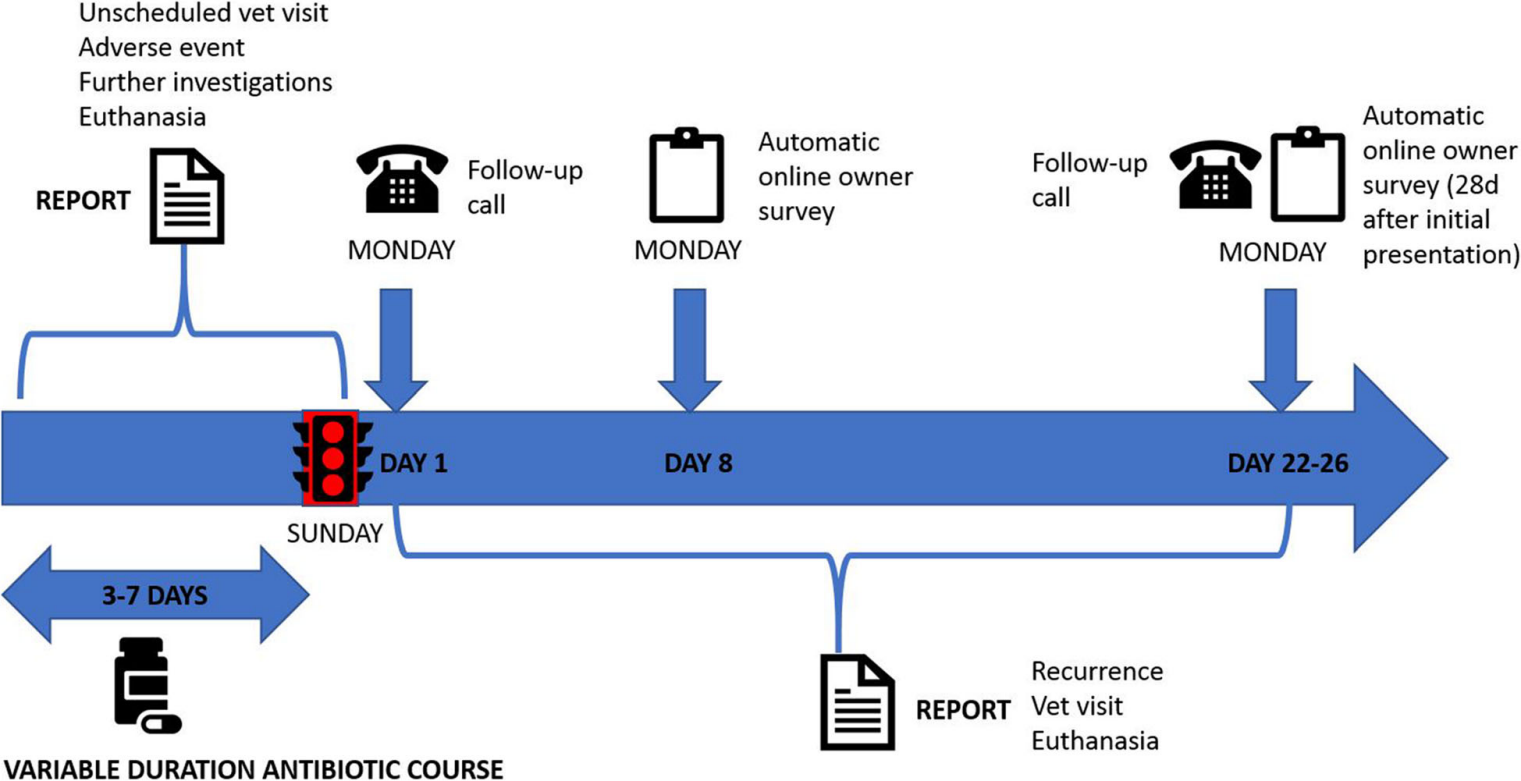
Infographic provided to participating veterinarians outlining key time points in the study

Each dog will start antimicrobial treatment immediately and the treatment will be continued twice daily until the next Sunday evening (two treatments to be administered on the Sunday). Antimicrobial treatment will be stopped on Sunday evening (Table 2 and Fig. 1).

**Table 2:** Schedule of Trial Procedures

Procedure	Day of presentation	Sunday after presentation	Monday after presentation	7 days after completion antimicrobials	28 days after initial presentation
Physical examination	X				
Antimicrobial treatment	X				
Stop antimicrobial treatment		X			
Telephone/in person follow up			X		X
Automated owner survey				X	X

### Monday after presentation

All owners should update the case clinician (or a colleague) on the Monday (Day 1) having completed the variable duration antimicrobial treatment course the day before. Follow-up communication should be completed on the specified day and repeat efforts should be made on the Tuesday to contact any owners that have not provided an update. The participating veterinarian should complete the relevant study form on the electronic data capture platform detailing the adherence to the treatment course (number of doses misses), resolution or persistence of clinical signs and any further treatment administered or investigations performed. Clinical resolution will be based on the absence of lower urinary tract signs (haematuria, pollakiuria or dysuria/stranguria).

Any dogs with persistence of any of the lower urinary tract signs will be managed at the discretion of the participating veterinarian. In these cases the decision for further management will be classified as either extension of amoxicillin-clavulanate, prescription of a different antimicrobial or discontinuation of antimicrobial therapy. Further diagnostic investigations performed at this stage will also be recorded; dogs that subsequently have a non-bacterial cause for the clinical signs identified will be censored from certain data analyses. Any additional treatment will be provided until the clinical signs are controlled, or additional investigations undertaken, as appropriate for the individual case and under the supervision of the case clinician.

### Additional follow-up

Online surveys will be automatically sent to owners 7 days after completion of the antimicrobial course (short-term follow up) and 28 days after initial presentation (to measure recurrence). These short surveys will confirm timing of resolution of clinical signs and whether the owner has observed any recurrence of lower urinary tract signs since completion of antimicrobial treatment. The data may also be inputted by the case clinician after speaking to the owner by telephone or consulting with them in person if the owner is unable to complete the survey themselves.

A study form will also be completed by the case veterinarian 28 days after initial presentation. This will record any re-presentation of the dog to the practice and detail any additional investigations that have been performed or treatments prescribed.

### Unexpected events

In the event of unexpected complications, owners will be able to contact their veterinary practice directly at any time and make use of their normal 24 h emergency care provision. The electronic data capture platform includes the facility to input details of unscheduled events including presentation for clinical deterioration, potential adverse reactions associated with the antimicrobial treatment, euthanasia, death or consultation for another unrelated condition.

### Primary and secondary objectives (Table 3)

**Table 3:** Objectives and Outcome Measures

Objectives	Outcome Measures	Time Point(s) of evaluation of this outcome measure (if applicable)
**Primary Objective** To compare disease-related outcome measures in dogs with presumed urinary tract infection treated with different duration (3, 4, 5, 6 or 7 day) regimens of amoxicillin/clavulanate. Find the shortest duration that is non-inferior to a duration of 7 days:	Optimal treatment duration in terms of proportion of dogs with resolution of lower urinary tract clinical signs as reported by the owner. Optimal is here defined as the shortest duration that is non-inferior to a duration of 7 days. The non-inferiority margin is set at 10 % [36].	1 day post treatment completion
**Secondary Objectives** To compare recurrence rates within the 4 weeks of initial presentation after completion of different duration (3, 4, 5, 6 or 7 day) regimens of amoxicillin/clavulanate	Proportion of dogs developing recurrence of lower urinary tract signs within 4 weeks of initial presentation	28 days after initial presentation
**Exploratory Objectives** In cases where urine culture is performed (not compulsory), to compare among groups:	Percentage of culture positive pre-treatment samples Percentage where bacteriological cure achieved	All

The primary, secondary and exploratory objectives will be followed to compare the efficacy of shorter durations (3,4,5,6) with the standard duration of 7 days of antimicrobial treatment for the management of presumed UTI.

Neither owners nor participating veterinarians will be blinded to the duration of antimicrobial treatment for that patient. The veterinarian prescribing the antimicrobial (or a colleague at the same practice) will also be responsible for follow-up appointments and assessing clinical signs to determine response to treatment. Blinding of technical analysis personnel to treatment groups will be conducted through the electronic data capture platform as necessary. Un-blinding of the co-investigators will occur following completion of the primary statistical analysis.

### Data analysis

After study completion, the data will be collated, and statistical analysis will be performed using R (Vienna, Austria [gamlss package]) and Prism® (GraphPad Software, San Diego, USA).

Dogs will be grouped according to the number of days of treatment prescribed (3, 4, 5, 6 or 7 days). Comparison of these groups at baseline will be performed to look for any potential confounding factors that could bias the results (e.g. different durations of clinical signs prior consultation). Differences in group composition will also be verified for age, neuter status, previous history of UTIs and presence of chronic kidney disease. For continuous parameters, descriptive statistics will be calculated and tabulated for each group. If data are normally distributed, the mean and standard deviation will be calculated, while median and interquartile range will be cited for skewed data. Comparisons between the groups will be made using match-paired Student’s t-tests for parametric data and Wilcoxon matched-pairs signed rank test for non-parametric data.

The primary and secondary analyses will be analysed by estimating duration-response curves using a fractional polynomial logistic model with duration as the only covariate and two polynomial terms [35, 36]. Subsequently, the optimal duration is estimated from this curve by finding the minimum duration D* corresponding to a cure rate that is still within the pre-specified margin of 10 % of the efficacy of the longest duration of 7 days. The optimal duration will be estimated from the duration-response curves by bootstrapping – resample 1000 times the observed dataset with replacement – to directly estimate the optimal duration in each bootstrap, where the optimal duration is defined as the shortest duration that falls within the non-inferiority margin of 10 % (risk difference) of the efficacy of the maximum duration of 7 days. A bootstrap 95 % confidence interval will be constructed from the bootstrap mean estimate and its standard error. The recommended duration would be the shortest duration that is larger than the estimated upper limit of the 95 % confidence interval [36]. This methodology of using the duration-response curves has been shown to have good inferential properties (type I and II error) in extensive simulations [36].

### Sensitivity analyses

Because there is a perfect correlation between day of presentation and assigned duration, results may be confounded by systematic differences in severity of illness when presenting just before or after the weekend compared to the rest of the week. If severity of illness would be worse on Monday because owners wait until the weekend has passed, one could erroneously chose a duration that is too short as optimal. We will explore the sensitivity of our results to such a weekend effect by exploring how strong and common such an unmeasured severity-of-illness confounder would have to be to draw the wrong conclusion about the optimal duration. Additional covariates besides the time since onset of first sign(s) will be considered for inclusion in the model in sensitivity analysis, including concurrent clinical signs (haematuria, pollakiuria and dysuria/stranguria).

Acknowledging that not all veterinarians would agree on what the minimum treatment efficacy they would find acceptable for shorter than the maximum duration of 7 days – a pre-specified margin of 10 % in our main analysis – we will explore for a range of margins that could be deemed acceptable (range 0–20 %) to determine what the optimal duration would be. We will estimate 95 % confidence intervals around the difference in the response between each duration (3, 4, 5, and 6 days) and the longest duration (7 days) using bootstrapping. This will subsequently be visualized by plotting the duration on the x-axis and the estimated difference and corresponding confidence intervals in response on the y-axis as done in Figure 4 in Quartagno et al. This allows a comparison with any (duration-specific) non-inferiority margin.

### Sample size calculation

**Fig. 2:**
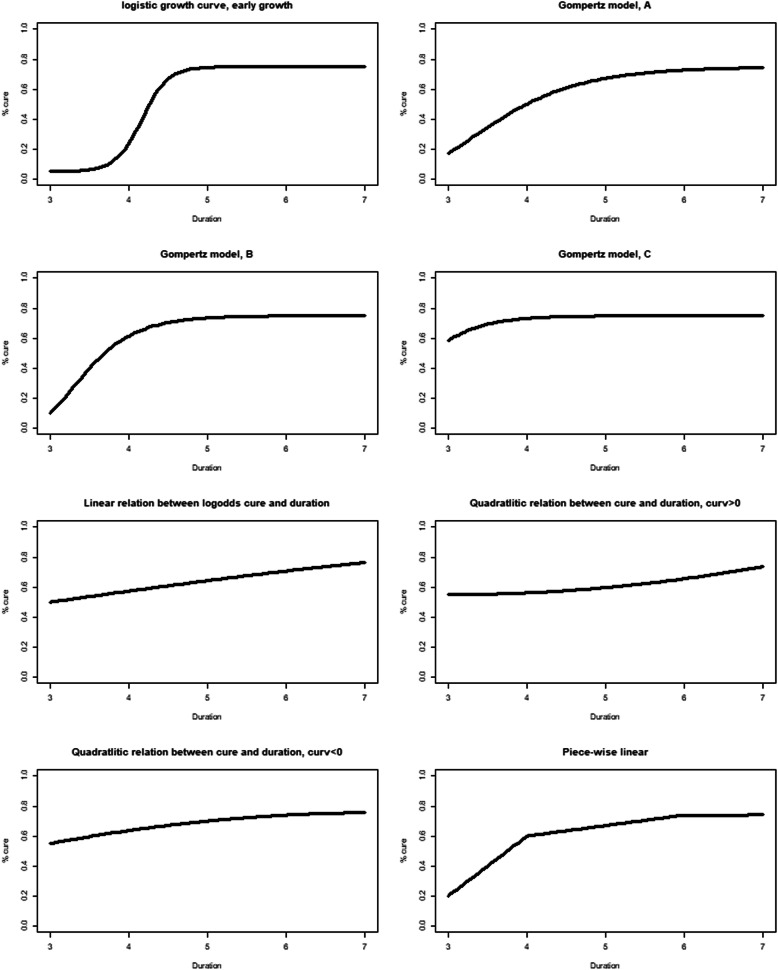
Hypothetical duration response curves to guide sample size calculations.

Sample size calculations were performed using simulations by modifying code that was developed and shared by Quartagno et al. [35] Several hypothetical duration-response curves (Fig. 2) were considered to guide the sample size calculations. To achieve 95 % of simulated trials leading to a duration-response curve whose error in the estimation of the probability of clinical cure was under 5 % for most hypothetical curves (95th percentile scaled area between curves (sABC) < 0.05) an effective sample size of 700 would be required (this figure would also be sufficient for comparison of recurrence data). However, it must be remembered that not all dogs enrolled within this study will have a UTI. Given the stringent inclusion criteria it is estimated that the percentage of dogs with a genuine UTI in this study will be 75 %. Therefore, without any dropouts the required sample size would be 700 / 0.75 = 934 dogs. With a non-inferiority margin of 10 % and an effective sample size of 700 participants a fixed-2 fractional polynomial model demonstrated robustness against a range of duration-response curves in simulations in terms of absolute error (< 5 % across most scenarios), acceptable power (> 90 % across most scenarios) and Type-I error (< 0.025 across most scenarios) [35, 36].

An effective sample size of 650 dogs still achieves a sABC < 0.05 for the Gompertz model C requiring recruitment of 650 / 0.75 = 867 dogs. Assuming that the majority of UTI cases that respond to antimicrobial therapy should do so within 48 h, the Gompertz model C would seem a priori the most likely true duration-response curve. Nevertheless, the use of a flexible fractional polynomial will ensure that other underlying shapes of the duration-response curve can be adequately estimated. In either case a projected sample size of nearly 900 dogs is ambitious. Interim analysis is proposed upon enrollment of 33 % cases (300 cases), or after 2 years of recruitment, whichever event comes sooner.

## Discussion

While several previous studies have compared defined treatment durations of different antimicrobial treatments, the approach outlined here is novel in (veterinary) medicine. By assigning patients to a number of duration arms and estimating a duration-response curve, the optimal antibiotic treatment duration can be estimated. This has several advantages compared to more traditional comparisons of a control duration and an often arbitrarily chosen shorter duration, which may well be not the optimal duration. For example, if 3 days is not non-inferior to 7 days, but 4 days is non-inferior to 7 days, the optimal duration identified by a standard 3 vs. 7 days non-inferiority trial would be 7 days, while a multi-arm DURATION design would identify 4 days as the optimal duration. By evaluating multiple durations in one trial one avoids erosion of efficacy from testing for non-inferiority for increasingly shorter durations in sequential trials with updated control durations, thereby avoiding the so-called ‘bio-creep’ [36, 39]. Furthermore, the design maintains good properties against a wide range of duration-response curves, while standard non-inferiority trials are vulnerable to losing power or interpretability if a single design parameter has been misspecified [36].

By estimating the optimal treatment duration for presumptive UTI, this study should support veterinarians to determine course lengths for similar presentations and ensure optimal antimicrobial use for a common canine affliction. The abbreviated name for this study – stop on Sunday (SOS), highlights the method of assignment of different treatment durations. All initial antimicrobial courses will be prescribed to complete on the Sunday evening after presentation. Therefore, dogs presenting on Monday will receive a 7-day antimicrobial course, those on Tuesday will receive 6 days’ worth of treatment and so forth. This per diem method of randomization was selected to facilitate effective safety-netting for the patient. By synchronizing case follow-up to a Monday, within 24 h of completion of the initial antimicrobial course, the participating veterinarian will have the opportunity to re-assess the case and enact appropriate diagnostic or therapeutic steps appropriate to the reported clinical response.

It may be argued that the day of presentation is not a genuine random variable; confounding factors could contribute to an uneven distribution of case presentations across the working week both in terms of the number of cases and case-severity. For example, owners may delay presentation over a weekend (reduced appointment availability) or conversely, present sooner, in anticipation of the weekend (same reasoning). Quantification of such factors is difficult and this methodology represents a limitation to the study, although measures of disease severity including separately the duration of pollakiuria, haematuria and dysuria/stranguria and the presence/absence of additional systemic clinical signs will be recorded and compared. Sensitivity analysis will be presented that will explore how large such effects should be to prompt the wrong inference about optimal treatment duration. However, this approach to randomisation does confer 2 notable advantages. Firstly, early outcome reporting on a Monday optimises the opportunity to adjust patient management protecting patient safety. Secondly, the process of group allocation is rapid and straightforward avoiding unnecessary delays during the consultation.

Consistent with National and International guideline documents, submission of urine culture is recommended as part of this study protocol. However, it has not been made a mandatory inclusion criterion. Empiric therapy without confirmation of diagnosis risks aggravating antimicrobial resistance [40]; any decision to treat must be based on a strong index of clinical suspicion. How confident one needs to be to justify empiric antimicrobial use remains a controversial topic. A threshold of 70 % probability of infection may be regarded as sufficient based on recommendations for management of uncomplicated cystitis in women [41, 42]. Accurate patient selection in this context is therefore crucial to minimise inappropriate treatment. The pre-test probability of UTI has been previously investigated in dogs [15] and people [43, 44]. Although confirmed bacterial infection was only found in 46 to 65 % of dogs presenting with one or more lower urinary tract signs [13–15, 45], by focusing on a narrow, increased-risk population (exclusively female dogs), the positive predictive value of each lower urinary tract sign should be greater than the values described for all dogs. The ultimate decision to enrol any dog requires an intention of the participating veterinarian to prescribe antimicrobial therapy. We estimate that 75 % of dogs treated on this basis will have a bacterial UTI.

The primary outcome of this study (clinical cure by the end of the treatment course) is dependent on the short term clinical cure rates for female dogs with sporadic cystitis treated with amoxicillin-clavulanate, previously found to be between 70 and 88 % [26, 27]. A clinical cure rate of 75 % for this study population has been assumed. For the secondary outcome, (recurrence rates within 3 weeks of completion of treatment), a recurrence rate of 10 % has been extrapolated [27]. Limited data is available to support this assumption.

In-vitro susceptibility to amoxicillin-clavulanate was found in 83 % of isolates from uncomplicated canine UTIs [24]. Even greater in-vivo efficacy may be anticipated as amoxicillin is concentrated in urine. Although greater in-vitro susceptibility to trimethoprim-sulphamethoxazole was found (90.3 %) [24]; authorised preparations of this antimicrobial are not currently commercially available in the UK. Culture and susceptibility testing is particularly important when antimicrobial therapy has previously been administered; in vitro resistance was higher in dogs treated in the last 30 days with antimicrobials [24]. This finding underlies the exclusion criteria relating to recent antimicrobial use in this study.

Recent guidance from the European Medicines Agency (EMA), prepared by the Antimicrobial Advice Ad Hoc Expert Group (AMEG), takes account of the need to use antimicrobials in animals versus the risk of antimicrobial resistance to public health [46]. Importantly, amoxicillin-clavulanate has been placed in Category C (Caution) while the aminopenicillins (e.g. amoxicillin) are Category D (Prudence). This reflects concerns that the overuse of beta-lactamase inhibitors will drive selection for resistance towards penicillins and cephalosporins, including the higher generation cephalosporins, in both Gram-negative bacteria (e.g. ESBL) and in staphylococci (e.g. MRSA). Further the ISCAID guidelines state that evidence supporting a need for clavulanic acid in the treatment of sporadic cystitis is not established[5]. Selection of a narrower spectrum antimicrobial (amoxicillin) would adhere more closely to key antimicrobial principles. However, where amoxicillin is not widely available or used (as the latest Veterinary Antimicrobial Resistance and Sales Surveillance (VARSS) data for antimicrobial use in the UK indicates [47]), the use of amoxicillin-clavulanate is reasonable [5]. Given the large sample size required in this study, amoxicillin-clavulanate was selected to facilitate maximal practice recruitment.

 This study has been purposefully designed to mirror current standard clinical practice; findings should, therefore, be applicable to a commonly-encountered situation supporting veterinarians in their decision making process. This study is the first in veterinary medicine to try to establish an optimal treatment duration for any infectious disorder and, if successful, will represent the largest participation trial undertaken to date. It is hoped that the results of this process will provide valuable information to guide antimicrobial therapy in the future and further antimicrobial stewardship as part of a wider one-health perspective.

## Supplementary Information

**Additional file 1.**

## Data Availability

The datasets used and/or analysed during the current study will be made available from the corresponding author on reasonable request. Datasets will be presented within a final manuscript and/or included as additional supporting files as appropriate. No identifying/confidential patient data will be shared.

## Abbreviations

AMR: Antimicrobial resistance
BSAVA: British Small Animal Veterinary Association
DALY: Disability-adjusted life-year
EMEA: European Agency for the Evaluation of Medicinal Products
FECAVA: Federation of European Companion Animal Veterinary Associations
GCP: Good Clinical Practice
HIV: Human immunodeficiency virus
MIC: Minimum inhibitory concentration
ISCAID: International Society for Companion Animal Infectious Diseases
SAMSoc: Small Animal Medicine Society
sABC: Scaled area between curves
SOS: Stop on Sunday
UTI: Urinary tract infection

## References

[1] Cassini A, Diaz Högberg L, Plachouras D (2019). Attributable deaths and disability-adjusted life-years caused by infections with antibiotic-resistant bacteria in the EU and the European Economic Area in 2015: a population-level modelling analysis. Lancet Infect Dis.

[2] Jessen LR, Damborg P, Spohr A (2018). Antimicrobial Use Guidelines for Companion Animal Practice [Antibiotikavejledning til familiedyr].

[3] Hillier A, Lloyd DH, Weese JS (2014). Guidelines for the diagnosis and antimicrobial therapy of canine superficial bacterial folliculitis (Antimicrobial Guidelines Working Group of the International Society for Companion Animal Infectious Diseases). Vet Dermatol.

[4] Lappin MR, Blondeau J, Boothe D (2017). Antimicrobial use guidelines for treatment of respiratory tract disease in dogs and cats: Antimicrobial Guidelines Working Group of the International Society for Companion Animal Infectious Diseases. J Vet Intern Med.

[5] Weese JS, Blondeau J, Boothe D (2019). International Society for companion animal infectious diseases (ISCAID) guidelines for the diagnosis and management of bacterial urinary tract infections in dogs and cats. Vet J.

[6] Lutz B, Lehner C, Schmitt K (2020). Antimicrobial prescriptions and adherence to prudent use guidelines for selected canine diseases in Switzerland in 2016. Vet Rec Open.

[7] Rantala M, Huovinen P, Holso K, Lilas A, Kaartinen L (2004). Survey of condition-based prescribing of antimicrobial drugs for dogs at a veterinary teaching hospital. Vet Rec.

[8] Gómez-Poveda B, Moreno MA (2018). Antimicrobial Prescriptions for Dogs in the Capital of Spain. Front Vet Sci.

[9] De Briyne N, Atkinson J, Pokludová L, Borriello SP (2014). Antimicrobials used most commonly to treat animals in Europe. Vet Rec.

[10] Ling GV (1984). Therapeutic strategies involving antimicrobial treatment of the canine urinary tract. J Am Vet Med Assoc.

[11] Adamama-Moraitou KK, Pardali D, Prassinos NN (2017). Evaluation of dogs with macroscopic haematuria: a retrospective study of 162 cases (2003–2010). N Z Vet J.

[12] KivistÖ A-K, Vasenius H, Sandholm M (1977). Canine bacteruria. J Small Anim Pract.

[13] Sørensen TM, Bjørnvad CR, Cordoba G (2018). Effects of diagnostic work-up on medical decision-making for canine urinary tract infection: an observational study in Danish small animal practices. J Vet Intern Med.

[14] Windahl U, Holst BS, Nyman A (2014). Characterisation of bacterial growth and antimicrobial susceptibility patterns in canine urinary tract infections. BMC Vet Res.

[15] Sørensen TM, Holmslykke M, Nordlund M, Siersma V, Jessen LR (2019). Pre-test probability of urinary tract infection in dogs with clinical signs of lower urinary tract disease. Vet J.

[16] Weese JS, Blondeau JM, Boothe D, et al. Antimicrobial use guidelines for treatment of urinary tract disease in dogs and cats: Antimicrobial guidelines working group of the international society for companion animal infectious diseases. Vet Med Int. 2011;2011(4):1–9. 10.4061/2011/263768 PMC3134992 21776346

[17] Ironmonger D, Edeghere O, Gossain S, Hawkey (2016). PM Use of antimicrobial resistance information and prescribing guidance for management of urinary tract infections: survey of general practitioners in the West Midlands. BMC Infect Dis.

[18] Ganzeboom KMJ, Uijen AA, Teunissen DTAM, Assendelft WJJ, Peters HJG, Hautvast JLA, Van Jaarsveld CHM (2019). Urine cultures and antimicrobials for urinary tract infections in Dutch general practice. Prim Health Care Res Dev.

[19] Spek M, Cals JWL, Oudhuis GJ (2020). Workload, diagnostic work-up and treatment of urinary tract infections in adults during out-of-hours primary care: a retrospective cohort study. BMC Fam Pract.

[20] Burke S, Black V, Sánchez-Vizcaíno F, Radford A, Hibbert A, Tasker S (2016). Use of cefovecin in a UK population of cats attending first-opinion practices as recorded in electronic health records. J Feline Med Surg.

[21] European Surveillance of Veterinary Antimicrobial Consumption (ESVAC) report. Sales of veterinary antimicrobial agents in 31 European countries in 2018. 2020. https://www.ema.europa.eu/en/documents/report/sales-veterinary-antimicrobial-agents-31-european-countries-2018-trends-2010-2018-tenth-esvac-report_en.pdf.

[22] Mateus A, Brodbelt DC, Barber N, Stärk (2011). KDC Antimicrobial usage in dogs and cats in first opinion veterinary practices in the UK. J Small Anim Pract.

[23] Hall JL, Holmes MA, Baines SJ (2013). Prevalence and antimicrobial resistance of canine urinary tract pathogens. Vet Rec.

[24] Wong C, Epstein SE, Westropp JL (2015). Antimicrobial susceptibility patterns in urinary tract infections in dogs (2010–2013). J Vet Intern Med.

[25] Smee N, Lloyd K, Grauer GF (2013). UTIs in Small Animal Patients: Part 2: Diagnosis, Treatment, and Complications. J Am Anim Hosp Assoc.

[26] Cotard JP, Gruet P, Pechereau D, Moreau P (1995). Comparative study of marbofloxacin and amoxicillin clavulanic acid in the treatment of urinary tract infections in dogs. J Small Anim Pract.

[27] Westropp JL, Sykes JE, Irom S (2012). Evaluation of the efficacy and safety of high dose short duration enrofloxacin treatment regimen for uncomplicated urinary tract infections in dogs. J Vet Intern Med.

[28] Clare S, Hartmann FA, Jooss M (2014). Short- and long-term cure rates of short-duration trimethoprim-sulfamethoxazole treatment in female dogs with uncomplicated bacterial cystitis. J Vet Intern Med.

[29] Jessen LR, Sørensen TM, Bjornvad CR, Nielsen SS, Guardabassi (2015). L Effect of antimicrobial treatment in canine and feline urinary tract infections: a systematic review. Vet J.

[30] Spellberg B. The New Antibiotic Mantra-"Shorter Is Better". JAMA Intern Med. 2016;176(9):1254-5. 10.1001/jamainternmed.2016.3646 PMC5233409 27455385

[31] Spellberg B, Rice LB (2019). Duration of antimicrobial therapy: shorter is better. Ann Intern Med.

[32] Pouwels KB, Hopkins S, Llewelyn MJ, Walker AS (2019). Duration of antibiotic treatment for common infections in English primary care: cross sectional analysis and comparison with guidelines. BMJ.

[33] Vaughn VM, Flanders SA, Snyder A (2019). Excess antimicrobial treatment duration and adverse events in patients hospitalized with pneumonia. A multihospital cohort study. Ann Intern Med.

[34] Pouwels KB, Yin M, Butler CC (2019). Optimising trial designs to identify appropriate antibiotic treatment durations. BMC Med.

[35] Quartagno M, Walker AS, Carpenter JR, Phillips PP, Parmar MK (2018). Rethinking non-inferiority: a practical trial design for optimising treatment duration. Clin Trails.

[36] Quartagno M, Carpenter JR, Walker AS, Clements M, Parmar MK (2020). The DURATIONS randomised trial design: Estimation targets, analysis methods and operating characteristics. Clin Trails.

[37] Allerton F, Kent A. How to approach urinary tract infections. Companion 2020.

[38] Electronic Data Capture (EDC). eCRF, ePRO, eCOA for clinical research | Castor. In: Castor EDC. https://www.castoredc.com/. Accessed 10 Dec 2020.

[39] MacFadden DR, Hanage WP (2019). Potential for Erosion of Efficacy in Noninferiority Trials of Decreasing Duration of Antibiotic Therapy. Clin Infect Dis.

[40] Butler CC, Dunstan F, Heginbothom M, Mason B, Roberts Z, Hillier S, Howe R, Palmer S, Howard A (2007). Containing antibiotic resistance: decreased antibiotic-resistant coliform urinary tract infections with reduction in antibiotic prescribing by general practices. Br J Gen Pract.

[41] McIsaac WJ, Moineddin R, Ross S (2007). Validation of a decision aid to assist physicians in reducing unnecessary antibiotic drug use for acute cystitis. Arch Intern Med.

[42] McIsaac WJ, Moineddin R, Gagyor I, Mazzulli T (2017). External validation study of a clinical decision aid to reduce unnecessary antibiotic prescriptions in women with acute cystitis. BMC Fam Pract.

[43] Bent S, Nallamothu BK, Simel DL (2002). Does this woman have an acute uncomplicated urinary tract infection?. JAMA.

[44] Giesen LG, Cousins G, Dimitrov BD (2010). Predicting acute uncomplicated urinary tract infection in women: a systematic review of the diagnostic accuracy of symptoms and signs. BMC Fam Pract.

[45] Brloznik M, Sterk K, Zdovc I (2016). Prevalence and resistance patterns of canine uropathogens in regard to concurrent diseases. Berl Munch Tierarztl Wochenschr.

[46] EMA/AMEG, 2020. Categorisation of antibiotics in the European Union, https://www.ema.europa.eu/en/documents/report/categorisation-antibiotics-european-union-answer-request-european-commission-updating-scientific_en.pdf.

[47] Veterinary Antimicrobial Resistance and Sales. Surveillance Report UK-VARSS. 2019 (2020). https://assets.publishing.service.gov.uk/government/uploads/system/uploads/attachment_data/file/942772/FINAL_MASTER_VARSS_2019_Report__updated_Dec_2020_.pdf.

