# Supplementary material for: Prospective trial of different antimicrobial treatment durations for presumptive canine urinary tract infections
Source: BMC Vet Res. 2021 Sep 6;17:299. doi: 10.1186/s12917-021-02974-y (PMC8422737; doi:10.1186/s12917-021-02974-y)
Supplement: Supplementary file 1 — Additional file 1. [file 12917_2021_2974_MOESM1_ESM.zip › SOS Owner consent form FINAL.DOCX]

**Managing urinary tract infections – the STOP on SUNDAY trial**

**INFORMATION SHEET FOR OWNERS**

**Primary Investigators:**

^1^ Fergus Allerton MRCVS, ^1^Andrew Kent MRCVS, James Swann MRCVS, James Warland MRCVS, Julien Bazelle MRCVS, Andria Cauvin MRCVS and Sarah Caddy MRCVS

^1^ Willows Referral Service, Solihull, UK

[medicine@willows.uk.net](mailto:medicine@willows.uk.net)

Tel: 0121 712 7070

**Collaborative Investigators :**

***BACKGROUND***

Urinary tract infections are common in dogs with up to 1 in 3 dogs experiencing this condition during their lifetime. The majority of cases make a rapid recovery following treatment with antibiotics given by your vet. However, overuse of antibiotics can lead to development of antibiotic resistant infections that could affect animal and human health. Antibiotic stewardship (rational antibiotic use) helps preserve the efficacy of antibiotics for future pets and people.

Doctors have always advised patients to complete an antibiotic course. In recent years the duration of antibiotic treatments has been decreased to limit unnecessary administration. There is very little information in veterinary medicine as to the ideal length of treatment for managing urinary tract infections. The best evidence is extrapolated from human medicine.

***THE TRIAL***

The current standard antibiotic course prescribed for dogs with cystitis is typically between 3-7 days. The purpose of this trial is to establish the optimal duration of antibiotic treatment for urinary tract infection in female dogs. Your vet may also offer additional diagnostic tests including urinalysis and imaging although these tests are not a compulsory part of the trial.

The duration of antibiotic treatment will depend on the day your dog first visits the vet as all courses will finish on SUNDAY evening. We will compare the different treatment lengths to see if shorter courses are as effective as longer courses. Please contact your vet on the MONDAY after completion of the antibiotic course with an update. We will contact you again by email survey 1 week after completing antibiotics and after about 1 month to find out how your pet responded to the treatment.

Please ensure that you give all medication to your dog as recommended by your vet. If you have concerns about possible side effects (these are rare but may include vomiting, diarrhoea or the development of a rash), please contact your vet.

***ETHICAL Considerations***

Dogs enrolled in the study will be offered the same treatments that are normally used for treating dogs with urinary tract infections. The study has obtained ethical approval from the School of Veterinary Medicine and Science, University of Nottingham and the use of the antibiotic in this manner has been approved by the Veterinary Medicines Directorate. The potential benefit of the study to dogs with urinary tract infection is that we may be able to establish the optimal treatment length for this condition and limit unnecessary antibiotic use in the future. Owner information will be kept confidential, data will be linked to the individual by a unique ID and stored separately.

***Your Participation in the Study***

Participation is entirely voluntary and you will be able to withdraw your dog from study at any time, if you should wish to do so. Unwillingness to participate or withdrawal will not affect your dog’s care. If you are happy to help us with this study, please sign the authorisation form below.

**Managing urinary tract infections – the STOP on SUNDAY trial**

**OWNER’S CONSENT FORM**

I give permission (please tick as appropriate):

……………………………………. for my dog to participate in this treatment trial as described. I have read and understood the Owner Information Sheet and have been given the opportunity to ask questions and receive answers to my satisfaction.

…………………………………..… for any data collected about my dog to be used for the current study and future research purposes.

**PLEASE COMPLETE IN BLOCK LETTERS:**

**Owner’s Name:**  __________________

**Animal’s Name:**  __________________

**Case No:** __________________

**Owner’s Signature:**  __________________

**Owner’s email address:**  __________________

**Date:** __________________

**Vet’s Name:**  __________________
