# Supplementary material for: Prospective trial of different antimicrobial treatment durations for presumptive canine urinary tract infections
Source: BMC Vet Res. 2021 Sep 6;17:299. doi: 10.1186/s12917-021-02974-y (PMC8422737; doi:10.1186/s12917-021-02974-y)
Supplement: Supplementary file 1 — Additional file 1. [file 12917_2021_2974_MOESM1_ESM.zip › Steps of the SOS study.pdf]

Small Animal
MEDICINE SOCIETY
Solving diagnostic and therapeutic challenges together

STOP on Sunday (SOS) Urinary Tract Infection Trial

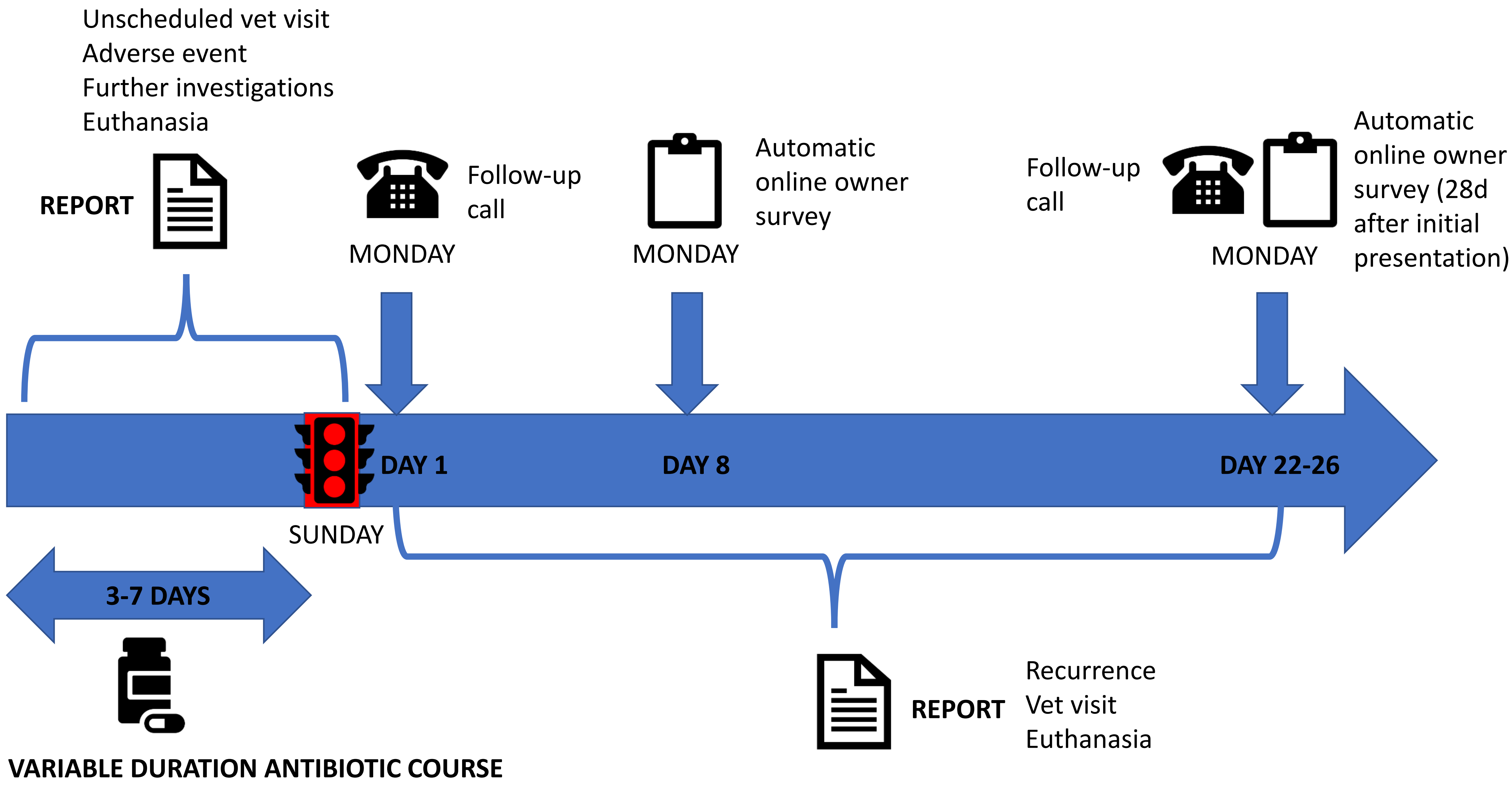
Unscheduled vet visit
Adverse event
Further investigations
Euthanasia
REPORT
Follow-up call
MONDAY
Automatic online owner survey
MONDAY
Follow-up call
Automatic online owner survey (28d after initial presentation)
MONDAY
DAY 1
DAY 8
DAY 22-26
SUNDAY
3-7 DAYS
VARIABLE DURATION ANTIBIOTIC COURSE
REPORT
Recurrence
Vet visit
Euthanasia

INCLUSION CRITERIA

- The owner is willing and able to give informed consent for participation in the study
- The dog is aged 6 months to 10 years
- The dog has a history of acute (< 7 days) pollakiuria, dysuria or haematuria
- The case clinician intends to prescribe antibiotics in the management of the case

EXCLUSION CRITERIA

- Detection of a non-bacterial cause of the clinical signs
- Suspicion of upper urinary tract involvement
  - presence of abdominal/lumbar pain
  - pyrexia (rectal temperature >39.5°C)
- Recurrent UTI
  - Previous UTI reported in the past 28 days
  - Three or more episodes of clinical bacterial cystitis in the last 12 months
  - Two or more episodes in the last 6 months
- Any dog on corticosteroid therapy, insulin, trilostane or thyroxine supplementation
- Dogs that have received antibiotics for any reason in the past 14 days.
- Known intolerance of amoxicillin/clavulanate
- Owners unable to administer medications q12 hours throughout treatment period

Steps of process (1st Visit)

- Confirm meets inclusion criteria and no exclusion criteria
- Obtain informed owner consent (sign consent forms)
- Prescribe amoxicillin-clavulanate
  - 12.5 mg/kg twice daily rounded up to the nearest possible tablet size or fraction that can be administered
  - Nº treatments reqd:

Day of presentation	Monday	Tuesday	Wednesday	Thursday	Friday
Nº treatments reqd. (am appt)	14	12	10	8	6
Nº treatments reqd. (pm appt)	13	11	9	7	5

- Enter details into Castor for 1st visit
- The owner email MUST be entered into CASTOR on the day of presentation

Steps of process (Monday after treatment)

- Antibiotic treatment will be completed Sunday evening (Day 0)
- Telephone contact with owner on the Monday (Day 1)
- Assess response to treatment
  - Record findings into Castor
  - Further treatment/diagnostics at the case clinician’s discretion

Steps of process (Owner surveys)

- Automatically emailed to owner on:
  - Day 8 (2nd Monday after completion of antibiotic course)
  - 28d after initial presentation (22-26 days after completion of antibiotic course)

- No input from case vet required
- Owners may receive email reminders to complete the surveys

Steps of process (3 week follow-up)

- Telephone contact with owner on the 4th Monday (Day 22)
- Assess response to treatment and any signs of recurrence
  - Record findings into Castor
  - Further treatment/diagnostics at the case clinician’s discretion

Steps of process (Unscheduled visits)

- In the following circumstances an extra report should be entered into the patient's Castor record:

  - Failure to respond or worsening of signs in the first week of treatment
  - Recurrence of lower urinary tract signs after completion ab course
  - Adverse event
  - Euthanasia/death
  - Performance of any diagnostics (urinalysis, radiography or ultrasound) outside of initial presentation
